# Amebic Liver Abscess With Intra-Biliary Rupture

**DOI:** 10.1155/1994/36160

**Published:** 1994

**Authors:** Md. Ibrarullah, Deepak K. Agarwal, Sanjay S. Baijal, Bhagwant R. Mittal, Vinay K. Kapoor

**Affiliations:** 1 Department of Surgical Gastroenterology Sanjay Gandhi Postgraduate Institute of Medical Sciences P. B.-375 Lucknow 226001 India; 2 Department of Gastroenterology Sanjay Gandhi Postgraduate Institute of Medical Sciences P. B.-375 Lucknow 226001 India; 3 Department of Radiology Sanjay Gandhi Postgraduate Institute of Medical Sciences P. B.-375 Lucknow 226001 India; 4 Department of Nuclear Medicine Sanjay Gandhi Postgraduate Institute of Medical Sciences P. B.-375 Lucknow 226001 India

## Abstract

The case of a large amebic liver abscess with an atypical presentation is reported. High output bile drainage persisted after ultrasound guided percutaneous catheter drainage because of a preexisting communication of the abscess with the right hepatic ductal system. The abscess was managed successfully by surgical evacuation and internal drainage into a defunctioned jejunal loop.


*HPB Surgery*, 1994, Vol. 7, pp. 305–313
Reprints available directly from the publisher
Photocopying permitted by license only

© 1994 Harwood Academic Publishers GmbH
Printed in the United States of America

### CASE REPORT

### AMEBIC LIVER ABSCESS WITH INTRA-BILIARY RUPTURE

MD. IBRARULLAH, DEEPAK K. AGARWAL*, SANJAY S. BAIJAL**, BHAGWANT R. MITTAL*** and VINAY K. KAPOOR

*Departments of Surgical Gastroenterology, Gastroenterology*, Radiology** and Nuclear Medicine***. Sanjay Gandhi Postgraduate Institute of Medical Sciences P.B.-375, Lucknow-226001, India*

*(Received 27 January 1993)*

The case of a large amebic liver abscess with an atypical presentation is reported. High output bile drainage persisted after ultrasound guided percutaneous catheter drainage because of a preexisting communication of the abscess with the right hepatic ductal system. The abscess was managed successfully by surgical evacuation and internal drainage into a defunctioned jejunal loop.

KEY WORDS: Liver abscess, amebic, bile fistula

### INTRODUCTION

Liver abscess is the commonest extra-intestinal manifestation of amebiasis. Its rupture into the biliary tree is a rare event, the exact incidence of which is not known. An abscess with this complication poses a management problem if routine external drainage is provided. One such amebic liver abscess which ruptured into the right hepatic ductal system is presented and its management discussed.

### CASE REPORT

A 24-year-old-male presented with a constant dull aching pain in the right upper abdomen, an intermittent low grade fever and anorexia for three months. During this period he recorded a weight loss of about 15kgs. On examination he was found to be deeply jaundiced and to have smooth tender hepatomegaly. His hemoglobin, total and differential leucocyte count were within normal limits. Liver function tests revealed serum bilirubin-11.9mg/dl (N 0.1–1.3) with a direct fraction of 9.6mg/dl, SGOT-68U/L (N 5–40), SGPT-68U/L (N 5–40), serum alkaline phosphatase 701U/

Address correspondence to: Vinay K Kapoor, Dept of Surgical Gastroenterology, Sanjay Gandhi Postgraduate Institute of Medical Sciences, P.B.-375, Lucknow-226001, India.

L (N 35–125). Abdominal ultrasonography (USG) showed a large hyperechoic mass nearly replacing the entire right lobe and extending into the left lobe. The intrahepatic biliary radicles were moderately dilated. A diagnosis of hepatocellular carcinoma was entertained. However, fine needle aspiration cytology of the lesion on three consecutive occasions was negative for malignant cells and serum alpha feto protein (AFP) was 4.58 IU/ml (N- up to 10 IU/ml). A repeat USG two weeks later showed some liquefaction in the lesion (Figure 1), raising the possibility of amebic liver abscess. Antiamebic antibodies (ELISA) at this stage were positive (64.8 EU/ml: N up to 40). The patient was started on oral metronidazole but did not show any response despite adequate treatment. He was subjected to USG guided percutaneous catheter drainage when a 9F pigtail catheter was introduced into the abscess cavity. The catheter initially drained brownish pus followed by frank bile. Bile output varied between 500–700 ml per day. Hepatobiliary scintigraphy using BULIDA showed preferential drainage of bile into the abscess cavity (Figure 2) and very little activity in the small intestine even after 24 hrs. Endoscopic retrograde cholangiography revealed extravasation of contrast medium into the abscess cavity from the main right hepatic ductal system (Figure 3). No reduction in cavity size could be demonstrated on USG even after four weeks and bile output remained constant i.e. 500–700 ml/day, although the patient became apyrexic and anicteric. Surgical exploration at this stage revealed a large abscess cavity almost replacing the entire right lobe of the liver. Since there was a thick fibrous wall surrounding the abscess cavity an opening was made on the inferior surface of the

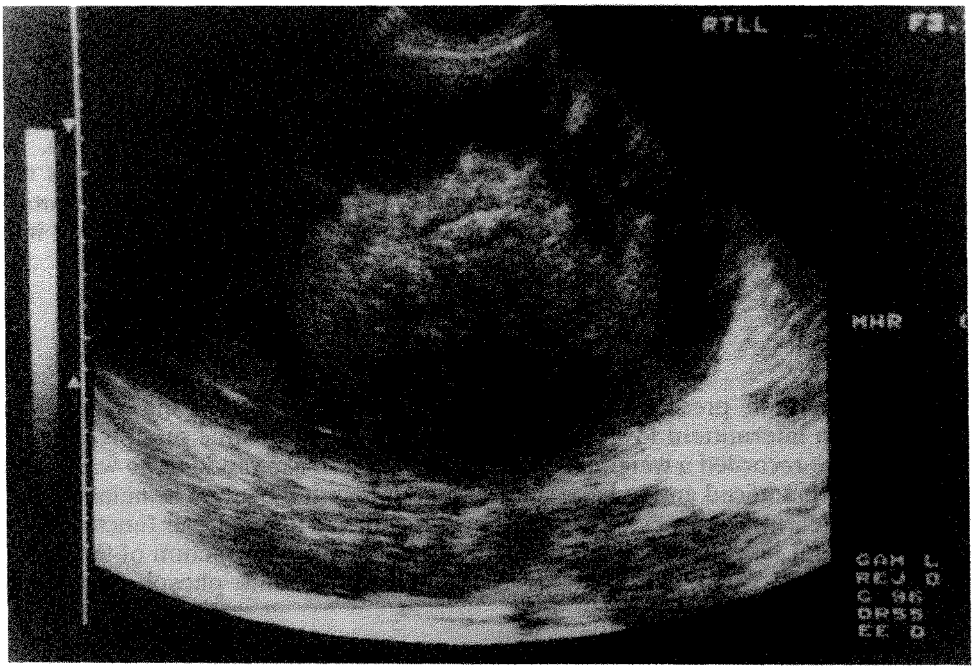

**Figure 1** Ultrasonogram of abdomen showing a predominantly hyperechoic space occupying lesion in the right lobe of the liver.

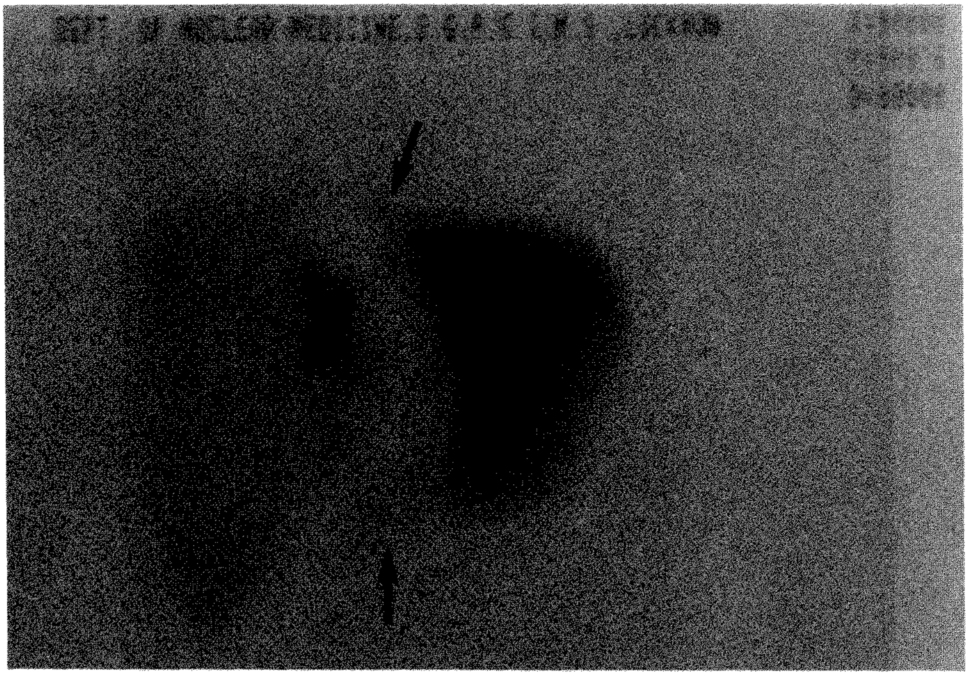

**Figure 2** Hepatobiliary scintigraphy. The right lobe of the liver is replaced by a large photopenic area (arrows) representing the abscess. Appearance of the tracer in the abscess cavity (arrow head) within 30 minutes of injection. Note, absence of excretion into small bowel.

right lobe of the liver and the abscess cavity was entered. Gentle debridement was carried out and a dependent internal drainage was provided by anastomosing a defunctioned loop of jejunum (Roux-en-Y) to this opening by interrupted sutures of non-absorbable material. The percutaneous external drainage was replaced by another wide bore catheter. The patient made an uneventful recovery. The external drainage catheter stopped draining from the 2nd postoperative day onwards and was removed on the 10th post operative day. Histology of the abscess wall revealed fibrocollangenous tissue, mixed inflammatory cells and occasional trophozoites of Entamoeba histolytica. A BULIDA scan done two weeks later revealed free flow of bile from the abscess cavity into the small bowel (Figure 4).

### DISCUSSION

Rupture into adjacent viscera or into pleural, peritoneal or pericardial cavity is a well known complication of amebic liver abscess. Rupture into the biliary tree is an uncommon entity and has been poorly addressed in the literature. The rarity of this complication is probably due to the resistance offered by the vasculobiliary sheath,

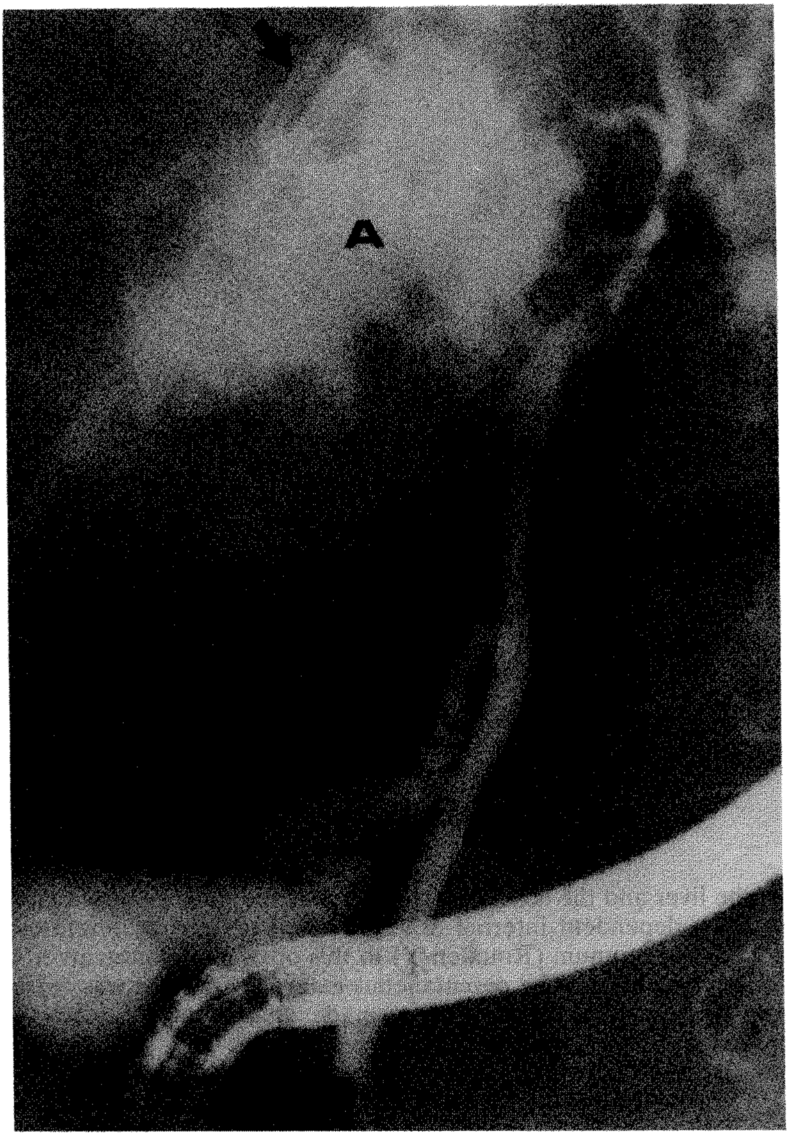

**Figure 3** Endoscopic retrograde cholangiopancreatography showing extravasation of contrast material into the abscess cavity (A). Percutaneous external drainage catheter in situ (arrow).

a tough fibrous tissue layer that surrounds the main or segmental portal structures: the bile duct, hepatic artery and portal vein.

The amebic liver abscess in the present case was initially misdiagnosed as hepatocellular carcinoma. The diagnostic error occurred because of the detection of a solid hyperechoic lesion on USG[1]. The correct diagnosis was, however

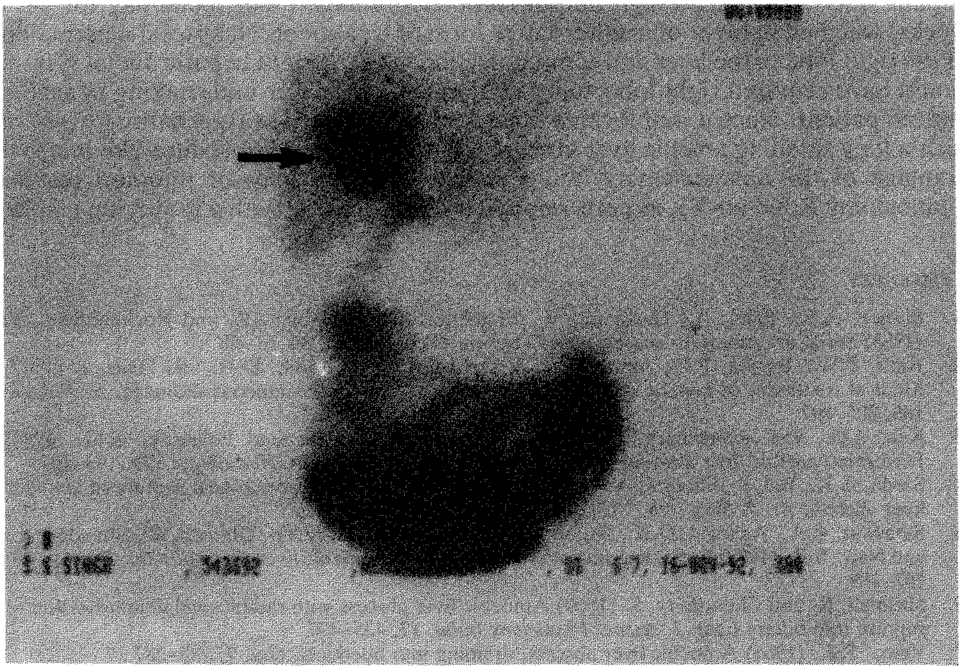

**Figure 4** Hepatobiliary scintography, two weeks after surgery. Free flow of tracer from the abscess cavity (arrow) into the anastomosed loop of jejunum.

established by positive amebic serology and biopsy of the wall of the abscess cavity after malignancy was ruled out by negative aspiration cytology and the normal AFP level.

The majority of amebic liver abscesses respond to conservative management[2]. Complicated abscess or those not responding to conservative treatment require interventional treatment in the form of needle aspiration, catheter drainage or surgical drainage. The current literature favors USG or CT guided percutaneous catheter drainage in the management of such abscesses[3–5]. The complicated abscesses dealt with in these reports are those with rupture into peritoneal or pleural cavity, bronchial tree or into adjacent viscera. No adequate management guidelines are available for those with rupture into the biliary tree. Some may respond to conservative treatment as has been outlined in a few anecdotal reports[6–8]. Powell *et al.* reported one such case presenting with hemobilia which was managed by operative ligation of the right hepatic artery[9]. Do *et al.* in a comparative study reported that 63% of liver abscesses with biliary communications can be successfully treated by prolonged catheter drainage[10]. The abscesses in their series were pyogenic in origin and had a smaller biliary communication since the majority were incidentally diagnosed on contrast radiography only.

Percutaneous catheter drainage in the present case was undertaken because of the large size of the abscess cavity, non-response to medical treatment and high serum bilirubin (11.9mg/dl). Communication with the major ductal system (right hepatic duct), prolonged high output catheter drainage (500–900ml/day for > 4 weeks) weighed in favor of an alternative form of treatment. Near total destruction of the right lobe of the liver and communication with the right hepatic ductal system would have required right lobectomy, undoubtedly a major undertaking in such a morbid patient. Since a well formed fibrous wall is almost always a natural event in such a long standing amebic liver abscess[11], a much simpler procedure like internal drainage of the abscess cavity into a defunctionalised loop of jejunum (cavito jejunostomy Roux-en-Y), as performed in the present case with a gratifying result.

### *References*

1. Simjee, A.E., Patel, A., Gathiram, V., Engelbrecht, H.E., Singh, K. and Rocknoodeen, F. (1985) Serial ultrasound in amoebic liver abscess. *Clin. Radiol.*, **36**, 61–68
2. Ralls, P.W., Barnes, P.F., Johnson, M.B. DeCock, K.M., Radin, D.R. and Halls, J. (1987) Medical treatment of hepatic amebic abscess: rare need for percutaneous drainage. *Radiology*, **165**, 805–807
3. Ken, J.G., van Sonnenberg, E., Gasola, G., Christenson, R. and Polansky, A.M. (1989) Perforated amebic liver abscess: successful percutaneous treatment. *Radiology*, **170**, 195–197
4. Sarda, A.K., Bal, S., Sharma, A.K. and Kapur, M.M. (1989) Intraperitoneal rupture of amoebic liver abscess. *Br. J. Surg.*, **76**, 202–203
5. Saraswat, V.A., Agarwal, D.K., Baijal, S.S., Roy, S., Choudhury, G., Dhiman, R., Bhandary, L. and Naik, S.R. (1992) Percutaneous catheter drainage of amoebic liver abscess. *Clin. Radiol.*, **45**, 187–189
6. Shrimali, R. and Choudhury, S. (1969) Amoebic liver abscess with radiological demonstration of rupture into biliary tract. *J. Assoc. Physicians India*, **17**, 435–437
7. Viana, R.L. (1966) Amoebic liver abscess, draining into bileducts. *Gut*, **7**, 285–287
8. Griffin, J., Jennings, C. and Owens, A. (1983) Hepatic amoebic abscess communicating with the biliary tree. *Br. J. Radiol.*, **56**, 887–890
9. Powell, S.J., Sutton, J.B. and Lautre, G. (1973) Hemobilia in amebic liver abscess. *S. Afr. Med. J.*, **47**, 1555–1557
10. Do, H., Lambiase, R.E., Deyoe, L., Cronan, J.J. and Dorfman, G.S. (1991) Percutaneous drainage of hepatic abscesses: comparison of results in abscesses with and without intrahepatic biliary communication. *A.J.R.*, **157**, 1209–1212
11. Baker, L.W. and Luvuno, F.M. (1988) Amoebiasis. In: Blumgart L.H. (ed.) *Surgery of the liver and biliary tract.* 1st ed, pp. 967–976. Edinburgh: Churchill Livingstone

*(Accepted by S. Bengmark 10 March 1993)*

### INVITED COMMENTARY

The authors of this article present a most unusual complication of amoebic abscess — a biliary fistula from the right main hepatic duct. After waiting for a period to see whether it would close spontaneously, they chose to deal with the problem by anastomosing a Roux loop to the wall of the abscess cavity. This relatively simple manoeuvre was successful in providing internal passage of bile, and at two weeks BULIDA scanning confirmed that bile flowed freely into the Roux loop.

An alternative solution might have been to remove most of the already destroyed right lobe and to ligate or oversew right hepatic duct. It might also have been possible to place a stent across the breach in the duct — a choice which would have disturbed the patient least of all. The authors decided against resection because of the morbid state of the patient — although they describe someone whose toxic state was well controlled by the percutaneous drainage. They also chose not to place a stent, and it is possible that the ductal injury made stent placement impossible. I cannot judge the precise place of injury from the ERCP reproduced in the article.

Their preferred alternative involved a "cavito-jejunostomy" with a defunctioned loop. The fistula continued to drain, but internally into the loop, as demonstrated by the BULIDA scan. Whether it will *continue* to drain internally is another matter. The cavity is likely to shrink in time, and as it does so the anastomosis may also shrink. This *may* not matter, but I have seen such anastomoses — to scar and granulation tissue — fail after months or years. If the residual cavity is small, there may not be any significant problem, but if it is of significant size, infection may occur within the stagnant collection.

It would be interesting to find out what happens to the patient reported in this paper *in the long term.* There is also a real need to find out what actually happens to anastomoses of this type, which do not involve the junction of normal tissues with a form and an enduring structure of their own. Perhaps someone who regularly uses a technique of this kind could produce some figures which might guide the rest of us.

Miles Little
Department of Surgery
Westmead Hospital
Westmead
NSW 2145, Australia

### INVITED COMMENTARY

With the increase in world travel, tropical diseases have become prevalent in parts of the world where previously they have been rare. Amoebiasis is a tropical disease caused by a protozoan parasite, Entamoeba histolytica, which can exist in the cystic or trophozoite forms. As trophozoites are easily destroyed by desiccation and gastric juice, transmission of the disease can only occur by swallowing cysts.

The common mode of transmission is by ingestion of water or vegetables contaminated by cysts. Direct faecal to oral transmission may occur under conditions of poor personal hygiene. A high proportion of the general population are cyst-passers in endemic areas. Even in advanced countries in the temperate zones as in the United States and the United Kingdom, surveys indicate a cyst-passer rate of 1 to 3%[1]. Thus, indigenous amoebiasis can occur without foreign travel in these countries, although the majority of cases still occur in immigrants or others who have returned from endemic areas[2]. There may be a long latent period between infection and the development of invasive amoebiasis, but this period may also be very short.

The most likely route for E. histolytica to reach the liver from the colon is via the portal blood stream. Microscopic examination by previous works has demonstrated multiple amoeba in the small inter-lobular branches of the portal vein[3]. Most patients with amoebic liver abscess have no evidence of intestinal disease at the time of clinical presentation, presumably the infection can remain dormant in the intestine for many years before spreading to the liver.

The diagnosis of amoebic liver abscess is based on the clinical and radiological features. It is confirmed by aspiration of pus from the liver, with demonstration of E. histolytica on microscopy, and by positive serology for amoebic antigens.

The majority of patients with amoebic liver abscess respond to medical treatment[4]. Metronidazole is highly effective and the recommended dose is 750 mg three times daily for ten days with or without a loading dose of 2.4 g followed by 1.2 g six-hourly[5]. A therapeutic response to metronidazole is a valuable aid to diagnosis, but this should be interpreted with caution because this drug is also effective against anaerobic organisms. There have been reports of failure of metronidazole to cure amoebic abscesses. If metronidazole is ineffective, choloroquine 500 mg daily for two to ten weeks, with or without dihydroemetine 1 to 1.5 mg per kg daily, given either intramuscularly or subcutaneously for ten days, should be added[6].

There is now less controversy over the indication for percutaneous needle aspiration of the abscess. As small abscesses respond very well to metronidazole, the only indication for aspiration in these cases is to confirm the diagnosis if this is in doubt. Even large abscesses have been shown to respond to drug treatment and needle aspiration should only be undertaken if there is evidence of an imminent danger of rupture into adjacent structures to avoid potentially fatal complications[7]. Surgical drainage should only be considered for patients who fail to respond to medical treatment and repeated needle aspiration, abscesses with impending rupture into the pericardium, abscesses which rupture into the peritoneum or a hollow viscus, and abscesses with secondary infection which are not controlled with antibiotics[8].

Complications of amoebic liver abscess include pleuro-pulmonary amoebiasis, peritonitis, cerebral and renal lesions and myocardial abscess[3,8]. Rupture of amoebic liver abscess into the biliary tree has been reported previously and all these patients responded to conservative management[3,9,10]. Powell and his associates reported a patient with haemobilia caused probably by rupture of the amoebic liver abscess into the bile duct and the blood vessel and the patient was managed by open surgery with ligation of the hepatic artery[11]. While amoebic liver abscess rupturing into the biliary system is so rare that the available medical literature can offer very little guidance on the management, treatment should follow established principles for treatment of complicated amoebic liver abscess. These, when properly applied as in the present case, can result in very satisfactory results.

W.Y. Lau
A.K.C. Li
Prince of Wales Hospital
Shatin
Hong Kong

### *References*

1. Juniper, K. (1978) Amoebiasis. *Clinics in Gastroenterology*, **7**, 3–29
2. Triger, D.R. (1978) Amoebic liver abscess in Wessex — a retrospective survey of 24 cases. *J. Trop. Med. Hyg.*, **81**, 54–59
3. Griffen, J., Jennings, C. and Owens, A. (1983) Hepatic amoebic abscess communicating with the biliary tree. *Br. J. Radiol.*, **56**, 887–890
4. Ralls, P.W., Barnes, P.F., Johnson, M.B., DcCock, K.M., Radin, D.R. and Halls, J. (1987) Medical treatment of hepatic amebic abscess: rare need for percutaneous drainage. *Radiology*, **165**, 805–807
5. Powell, S.J., Macleod, I., Wilmot, A.J., Elsdon-Dew, R. (1966) Metronidazole in amoebic dysentery and amoebic liver abscess. *Lancet*, **11**, 1329–1331
6. Krogsted, D.J., Spender, H.C. and Healy, G.R. (1978) Current concepts in parasitology. Amebiasis. *N. Engl. J. Med.*, **298**, 262–265
7. Sukov, R.J., Cohen, L.J. and Sample, W.F. (1980) Sonography of hepatic amoebic abscess. *American Journal of Roentgenology*, **134**, 911–915
8. Holdstock, G., Balasegaram, M., Millward-Sadler, G.H. and Wright, R. (1985) The liver in infection. Chapter 39, In: *Liver and Biliary Disease*. Ed: Wright Millward-Sadler Alberti Karran 2nd Edition. Bailliere Tindall, London, pp. 1104–1119
9. Shrimali, R. and Chondhurry, S. (1969) Amoebic liver abscess with radiological demonstration of rupture into biliary tract. *J. Assoc. Physicians India*, **17**, 435–437
10. Viana, R.L. (1966) Amoebic liver abscess draining into bile ducts. *Gut*, **7**, 285–287
11. Powell, S.J., Sutton, J.B. and Lautre, G. (1973) Hemobilia in amebic liver abscess. *S. Afr. Med. J.*, **47**, 1555–1557

